# Supplementary material for: Comparison of Genotype II African Swine Fever Virus Strain SY18 Challenge Models
Source: Viruses. 2023 Mar 27;15(4):858. doi: 10.3390/v15040858 (PMC10142125; doi:10.3390/v15040858)
Supplement: Supplementary file 1 [file viruses-15-00858-s001.zip › viruses-2251947-supplementary.pdf]

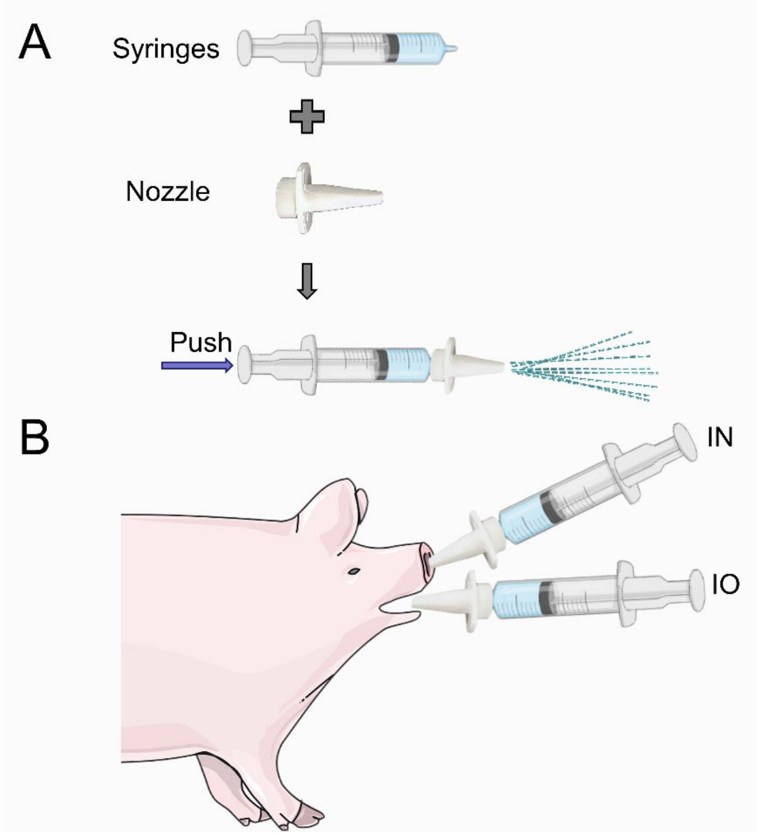

Figure S1: Intraoral and intranasal infection with ASFV SY18 using a nozzle. (A) Aerosol treatment of virus-containing liquids with nozzles; (B) Intraoral and intranasal infection

Table S1. Clinical scoring scale

Clinical Signs	lever	Score[a]
Rectal temperature	< 40.0	0
	40.0 ≤ to < 40.5	1
	40.5 ≤ 41	3
	> 41	5
Lost appetite	Reduced eating	1
	Only picking at food	3
	Not eating	6
Reluctance to stand	Lethargic	1
	Get up only when touched	3
	Remain ecumbent when touched	6
Wheezing/ coughing	Occasional	1
	Severe	3
Ocular discharge	Discharge around eyes	1
Enlarged joints	Mild joint swelling	1
	Severe swelling with difficulty walking	4
Skin Purpura	Redness of the skin	1
	Purple-black petechiae under the skin	3
Diarrhoea	With symptoms	1

Bloody diarrhoea	With symptoms	4

[a] Only the most severe one of each symptom was scored. Scores would not be accumulated to the next day.

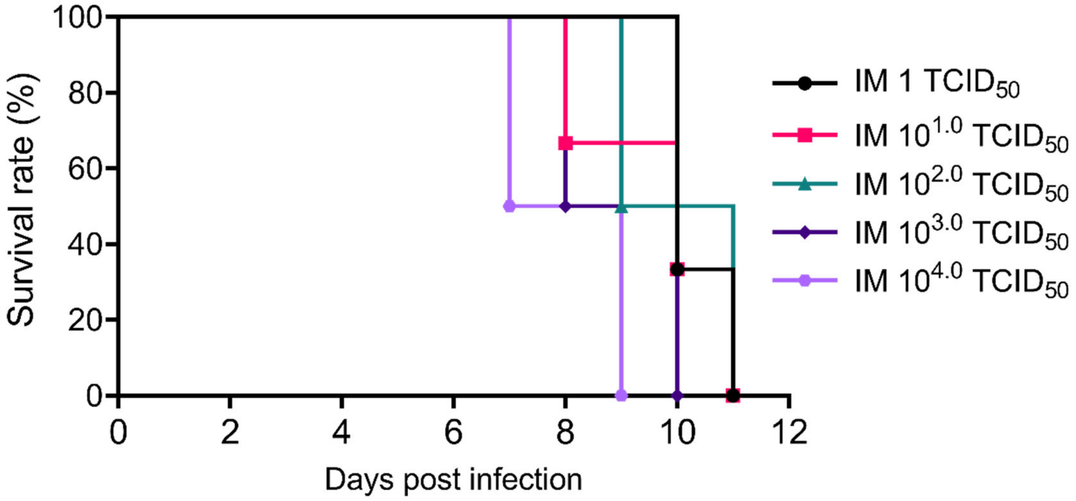

Figure S2: Survival rates of pigs infected with ASFV SY18 by intramuscular (IM) injection at doses of 1 $TCID_{50}$-$10^{4.0}$ $TCID_{50}$.
